# How duration and frequency influence horticultural therapy’s effect on depressive symptoms: evidence from a meta-analysis

**DOI:** 10.3389/fpsyg.2025.1575441

**Published:** 2025-05-16

**Authors:** Lin Chen, Youlong Sun, Yidian Pan, Ruqi Chen, Chang Liu

**Affiliations:** College of Landscape Architecture and Art, Fujian Agriculture and Forestry University, Fuzhou, Fujian, China

**Keywords:** horticultural therapy, depressive symptoms, duration and frequency, meta-analysis, landscape architecture

## Abstract

**Background:**

Horticultural therapy (HT) has been documented to significantly intervene in depressive symptoms, but the effect of temporal characteristics is unclear.

**Methods:**

11 databases were searched updated by 12th October 2024. 33 studies were included through quality assessment. A standardized mean difference (SMD) employing a random-effects model was used to assess the effect size of HT intervention for depressive symptoms, and the effect size was compared for different frequency, duration, session duration subgroups.

**Results:**

Overall, HT interventions for depressive symptoms were effective (SMD = −0.95). For intervention frequency, less than 3 times weekly (SMD = −1.21) was superior to 3 and more times weekly. For intervention duration, 5–8 weeks (SMD = −1.75) was superior to shorter (≤ 4 weeks) and longer (≥ 9 weeks) programs. For session duration, more than 60 min (SMD = −1.35) was superior to shorter ones. Optimal effects may emerge when implementing less than 3 HT sessions weekly, each lasting over 60 min, for a duration of 5–8 weeks.

**Conclusion:**

This study revealed the influence of temporal characteristics on the effectiveness of HT intervention for depressive symptoms. Since most of the included studies were conducted in Asia, the conclusions can better guide HT practices for Asian cultural groups.

**Systematic review registration:**

https://www.crd.york.ac.uk/PROSPERO/view/CRD42024523923, CRD42024523923.

## 1 Introduction

### 1.1 Depression and related interventions

Depression is a prevalent mental health disorder characterized by persistent low mood and a significant loss of interest or pleasure in activities, accompanied by a range of physical and psychological symptoms that can severely impair an individual’s daily functioning, including work, study, and social interactions (World Health Organization, 2019). According to the World Health Organization (WHO), depression is one of the leading causes of global disability and poor health. The prevalence of depression has been increasing steadily over recent years (Seok and Kim, 2024). Research indicates that approximately 5% of adults worldwide experience depression annually, with the peak incidence occurring around the age of 20 (Yang et al., 2024). Currently, the primary treatments for depression include pharmacotherapy and psychotherapy. Psychotherapy, including horticultural therapy (HT), is often recommended for mild-to-moderate cases owing to its minimal side effects and low shame (Farah et al., 2016; Seok and Kim, 2024).

### 1.2 Effectiveness of horticultural therapy on depressive symptoms

According to the Attention Restoration Theory and Biophilic Hypothesis, natural exposures, such as exposure to plants, have many mental health benefits, including stress reduction, cognitive restoration, and emotion regulation (Wilson, 1984; Kaplan and Kaplan, 1989; Meore et al., 2024). HT, a complementary therapy centered on plant exposure, has been empirically validated to improve mental health outcomes (Chan et al., 2017; Cipriani et al., 2017). By leveraging the therapeutic effects of the natural environment, HT reduces the stigma associated with traditional pharmacotherapy while promoting help-seeking behavior and mitigating the risk of chronic diseases (He et al., 2024). Consequently, HT has gained increasing recognition in recent years for its effectiveness in alleviating depressive symptoms. HT may improve depressive symptoms in non-hospitalized adults (Briggs et al., 2023). A meta-analysis study showed that HT was significantly effective in reducing depressive symptoms in older adults (Xu et al., 2023). In a meta-analysis of 35 randomized controlled trials (RCTs), researchers found that a short-term (≤3 months) HT program had a significant impact on depressive symptoms in patients with severe schizophrenia, whereas an HT program that lasted longer than 3 months had a moderate-to-large improvement effect on depressive symptoms in patients with moderate schizophrenia (Lee et al., 2024).

### 1.3 Deficiencies of temporal characteristics in horticultural therapy research

The temporal characteristics of HT intervention are key factors influencing therapeutic outcomes. These factors typically include intervention frequency, intervention duration, and session duration. In the context of psychotherapy for depressive symptoms, a study found that twice-weekly treatment frequency, whether cognitive behavioral therapy or interpersonal psychotherapy, had a more significant impact on depressive symptoms than once-weekly frequency (Bruijniks et al., 2020). Another study investigated the duration of antidepressant medication in patients with depression and its impact on relapse rates after discontinuation. The findings revealed that an initial active treatment period exceeding 3 months effectively reduced the risk of recurrence following discontinuation (Hu et al., 2024). In cases of major depressive disorder (MDD), antidepressants should usually be continued for a longer period of time after treatment of an acute episode to prevent relapse (Sorensen et al., 2022).

While specific studies on the temporal characteristics of HT for improving depressive symptoms are limited, previous research has provided valuable insights. A meta-analysis conducted by one study examined the effects of HT on stress relief and found that to achieve optimal improvements in physiological indicators, HT sessions should last 30–60 min once a week. For optimal psychological improvements, sessions lasting less than 30 min conducted 2–3 times per month were most effective. A cumulative intervention time of 100–500 min was determined to be the most effective for both physiological and psychological outcomes (Lu et al., 2023). Through subgroup analysis, another study revealed that 4–8 weeks of HT significantly improved depressive symptoms in elderly patients compared to the control group, while no significant differences were observed after 8 weeks (Xu et al., 2023). In a study on HT intervention for schizophrenia, longer weekly interventions led to better improvements in total and negative symptoms (Lee et al., 2024). Additionally, a study suggested that intervention frequencies of at least twice a week significantly enhanced cognitive function, with shorter intervention duration (<6 months) being more effective than longer ones (Wang et al., 2024).

### 1.4 Research question and purpose

This study aims to conduct a systematic review and meta-analysis to evaluate how the temporal characteristics of HT influence its treatment outcomes for depressive symptoms. The objectives include exploring (1) the effects of different intervention frequency of HT on improving depressive symptoms; (2) effects of intervention duration in HT on enhancing depressive symptoms; (3) session duration in HT on improving depressive symptoms. Meta-analysis serves as a fundamental method in evidence-based medical research, enabling us to draw statistically sound conclusions (Moher et al., 2009). Therefore, we conducted a meta-analysis to comprehensively analyze the therapeutic effects of HT on depressive symptoms by evaluating its timing aspects.

## 2 Methods

### 2.1 Search strategy

The systematic review was conducted in accordance with the Preferred Reporting Items for Systematic Reviews and Meta-Analyses (PRISMA) guidelines (Page et al., 2021a; Page et al., 2021b). This review was registered in the International Registry of Prospective Systematic Reviews (PROSPERO) with registration number CRD42024523923. In this study, we developed a search strategy based on the study population, intervention, control, outcome measures, and type of study (PICOS) framework. Two investigators independently searched multiple databases and resolved any disagreements through discussion or third-party adjudication. The English databases included PubMed, Embase, MEDLINE, Cochrane Library, CINAHL Plus with Full Text, PsycINFO, and Scopus, while the Chinese databases included China National Knowledge Infrastructure (CNKI), Wanfang Data, VIP Data, and China Biomedical Literature Database (CBM). Only Chinese literature was searched in the Chinese databases. There were no specific restrictions regarding the date range or the age and gender of the participants. The language restrictions were set to include both Chinese and English articles. The initial review covered articles from the inception of each database until May 5th, 2024, resulting in a total of 10,960 retrieved articles. The literature search was updated on October 12th, 2024, retrieving a total of 11,349 articles. The search strategy consisted of the following components, with each core element connected by the AND operator. Subject terms and free words were linked using OR. (1) Disease types: Depression (e.g., ‘Depressive Disorder’, depress*, melancholia*), (2) Interventions: Horticultural Therapy (e.g., horticult*, farm*, garden*), (3) Research methods: ‘Randomized Controlled Trials’, RCTs, ‘quasi-experimental studies’. Independent health information experts from external organizations reviewed the search terms to ensure the relevance and comprehensiveness of the strategy. Additionally, we examined the reference lists of previous reviews and key articles retrieved for relevant studies.

### 2.2 Inclusion criteria and exclusion criteria

The inclusion and exclusion criteria were based on the PICOS principles. The inclusion criteria comprised the following: (1) population: no restrictions; (2) interventions: HT; (3) comparison: no restrictions on control group; (4) study design: randomized controlled trials (RCTs) and quasi-experimental studies. Studies with unavailability of full-text literature, duplicate publications or data, studies of multiple interventions, inability to extract exact data directly for studies. Studies that do not include complete temporal characterization data (e.g., <3 times/week, <4 weeks, or >60 min), non-Chinese or English literature, abstracts, conference papers, books, and dissertations were excluded. The delineation of intervention frequency, duration, and session duration was based primarily on the temporal characteristics of the included studies as well as the characteristics of other meta-analyses related to HT (Lu et al., 2023; Xu et al., 2023) (The frequency data of some studies only includes the total duration and the total number of activities. In this study, the average number of activities per week was calculated.).

Current research on HT for depressive symptoms has primarily assessed the therapeutic efficacy of this approach using physiological and psychological indicators. This study specifically extracted and statistically analyzed relevant data pertaining to psychological indices. Psychological data were predominantly collected through standardized tests, such as the Geriatric Depression Scale Short Form(GDS-SF), Korean Version of the Geriatric Depression Scale Short Form(GDSSF-K), Beck Depression Inventory (BDI), Korean version of Beck Depression Inventory (K-BDI), Self-rating Depression Scale (SDS), Depression Anxiety and Stress Scale (DASS), Hamilton Depression Scale (HAMD), Center for Epidemiologic Studies Depression Scale (CES-D), Children’s Depression Inventory (CDI), and Korean Mental Health Screening Tool for Depression (MHS:D).

### 2.3 Study selection, data extraction and analysis

Two independent reviewers extracted the data to minimize bias. All records were systematically managed using EndNote X9. Discrepancies were resolved through discussion with a third reviewer until consensus was reached. Extracted variables included publication year, first author, country/region, sample size, participant ages, temporal characteristics (intervention frequency, intervention duration, session duration), environmental settings, and outcome measures. The continuous outcome calculation method was employed to extract the mean, standard deviation, and sample size from RCTs and quasi-experimental studies. In cases of discordance between textual and tabular data, precedence was given to textual descriptions unless clear evidence supported tabular accuracy.

### 2.4 Quality assessment

The Cochrane Risk of Bias Assessment Tool for Randomized Trials (ROB 2.0) (Higgins et al., 2011; Cumpston et al., 2019) was employed to evaluate RCTs, with the following domains assessed: randomization process, deviations from intended interventions, missing outcome data, outcome measurement, and selective reporting. Each domain was rated using three levels: low risk, some concerns, and high risk. For quasi-experimental studies, the Joanna Briggs Institute (JBI) critical appraisal tools (Peters et al., 2021; Barker et al., 2024) were utilized, requiring evaluators to judge each item as “yes,” “no,” “unclear,” or “not applicable.” Final inclusion/exclusion decisions were reached through panel discussions. The exclusion criteria were stipulated as follows: (1) quasi-experimental studies with >2 “No” responses on the JBI tools; (2) RCTs rated high risk as per the ROB 2.0 criteria.

### 2.5 Statistical analysis

Data analysis, processing, and graphical representation were conducted using RevMan 5.4 and Stata 18 software. The continuous outcome calculation method was employed to extract the mean, standard deviation, and confidence intervals from RCTs and quasi-experimental studies. This study primarily collected psychological data, with standardized tests (depression scales) serving as the main source of relevant information. Given the varying units of mental scales used in the included studies, we calculated the standardized mean difference (SMD) Hedges effect size. A negative Hedges effect indicated a significant reduction in depressive symptoms in the experimental group compared to the control group. Results were summarized using 95% confidence intervals (CIs). To assess heterogeneity across intervention effects in different studies, substantial heterogeneity was considered when *I*^2^ exceeded 50%. If *I*^2^ surpassed this threshold, sensitivity analysis and subgroup analysis were conducted to further investigate potential sources of heterogeneity, and meta-regression analysis was performed to aid accurate interpretation of study results. Additionally, a random-effects model was applied to minimize errors and obtain a comprehensive estimate of the effect size. Conversely, if *I*^2^ was <50%, a fixed-effects model was used for statistical analysis. Publication bias was assessed using funnel plots and Egger’s test owing to its potential impact on meta-analysis results from selective inclusion/exclusion criteria.

## 3 Results

### 3.1 Search outcomes

**Figure 1:**
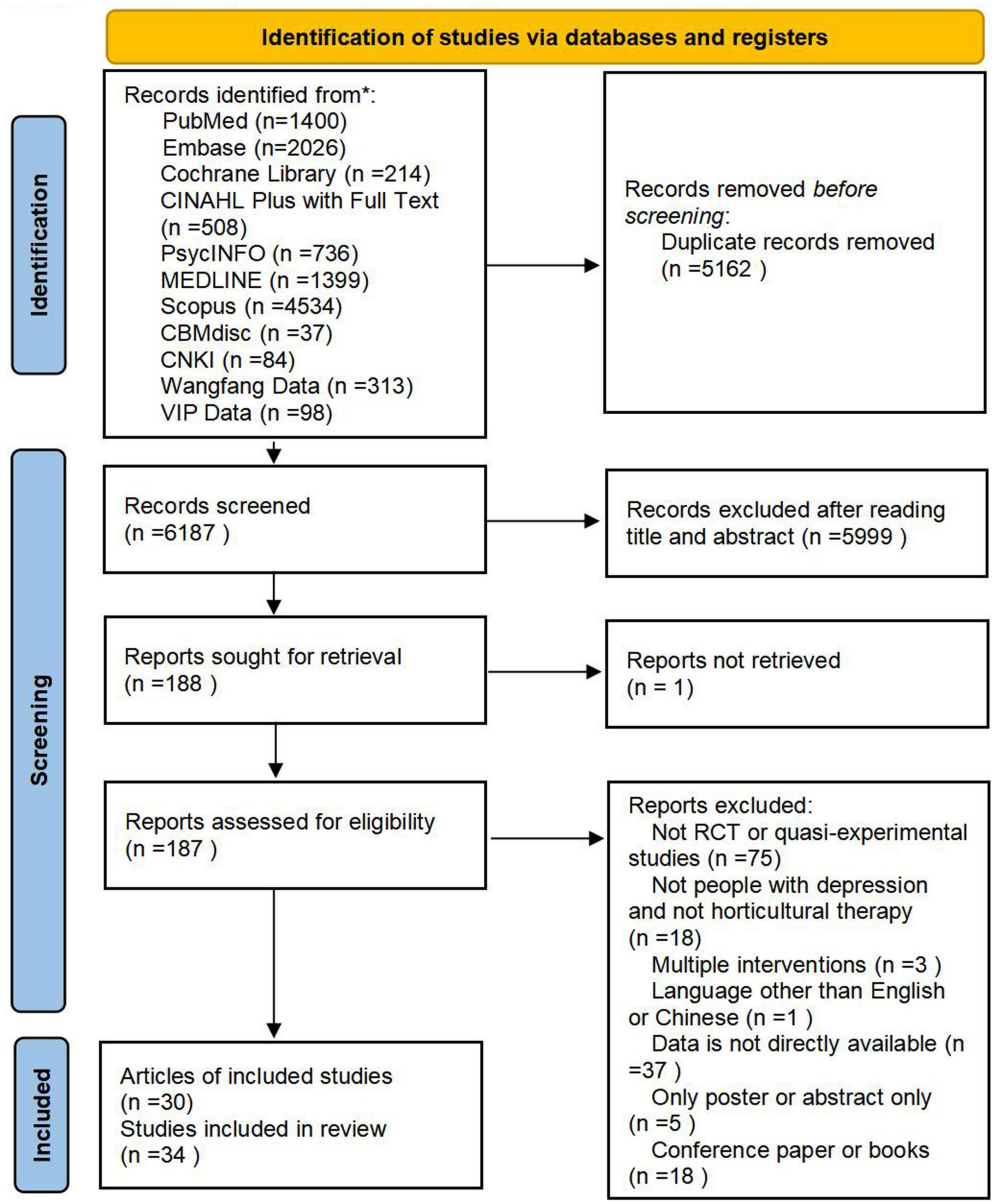
Preferred reporting items for systematic reviews and meta-analyses (PRISMA) flow diagram for search, selection, and identification process of this study.

**Figure 2:**
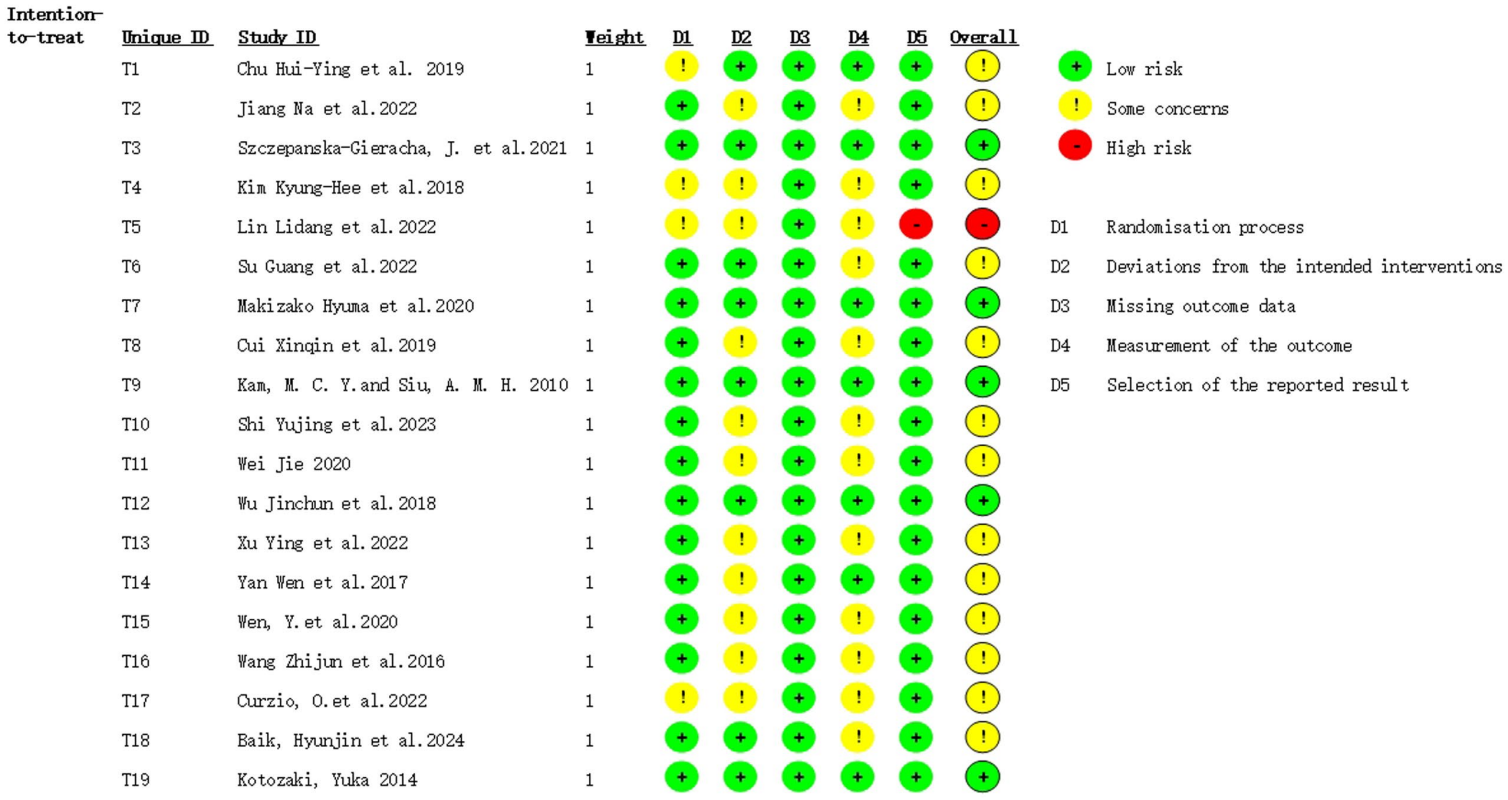
Summary of risk of bias in randomized controlled trials.

**Figure 3:**
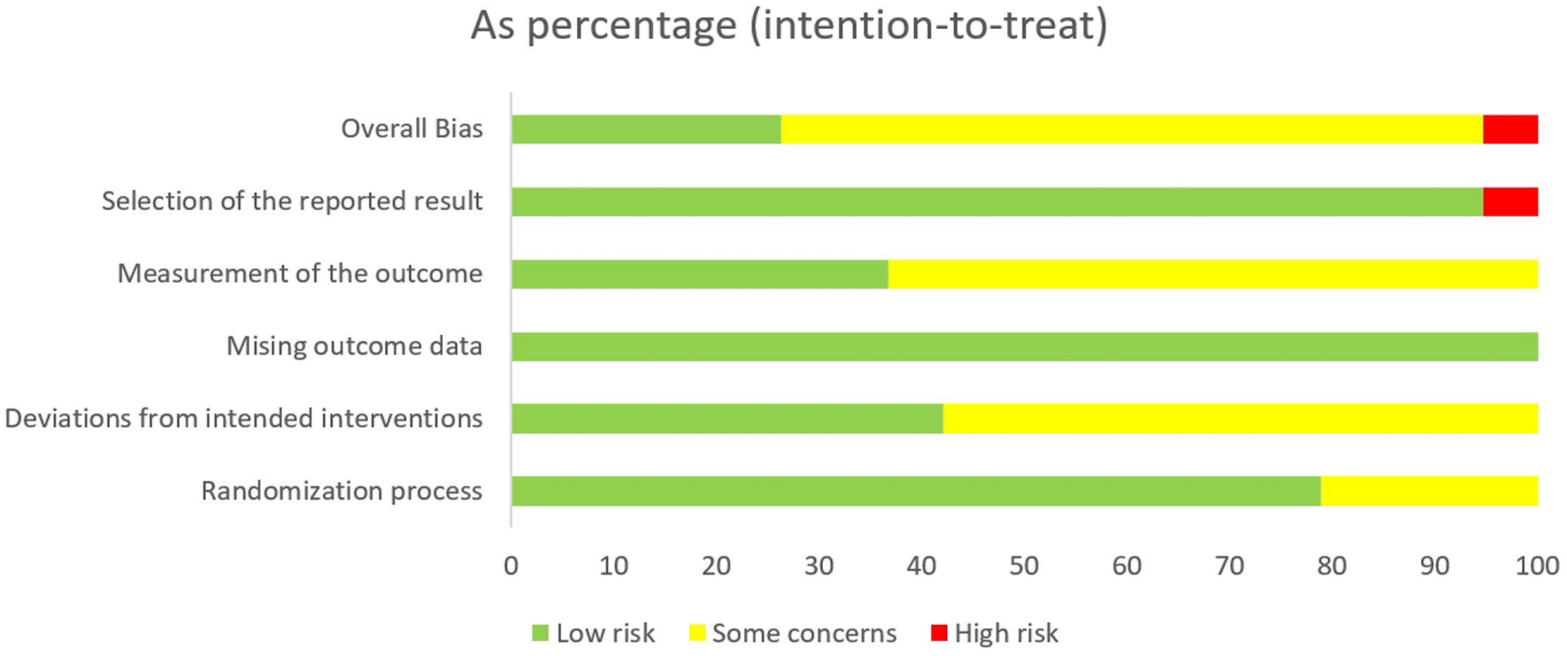
Risk of bias in randomized controlled trials.

As shown in Figure 1, the PRISMA flow chart indicates that 11,349 records were retrieved and screened. After excluding 5,162 duplicates and 5,999 irrelevant studies during title/abstract screening, 188 full-text articles underwent eligibility assessment. Studies were further excluded if they failed to meet predefined criteria or were unavailable in full text. Ultimately, this review included 30 articles encompassing 34 studies (Table 1). Following risk assessment, one study (Lin et al., 2022) was excluded for having an overall high risk of bias in RCTs (Figures 2, 3, Table 2). Regarding quasi-experimental studies, although all met the inclusion criteria, some exhibited specific methodological risks, such as the absence of control groups and baseline data imbalances (Table 2). Consequently, after applying the exclusion criteria, a meta-analysis was conducted using data from 33 studies (29 articles), including 21 RCTs and 12 quasi-experimental studies.

**Table 1:** Summary of characteristics of studies in current meta-analysis.

Study	Study design	Study site	Sample size	Ages means (SD) (E/C)	Intervention frequency (HT)	Session duration (HT)	Intervention duration (HT)	Intervention/control	Outcome measures
Chu et al. (2019)	RCT	China	75E/75C	79.2/77.9	Once-weekly	90 min	8 weeks	HT/RC	GDS-15
Jiang et al. (2022)	RCT	China	30E/30C	≥60	Once-weekly	90 min	8 weeks	RC with HT/RC	GDS-30
Szczepanska-Gieracha et al. (2021)	RCT	Poland	11E/12C	70.18 (4.87) / 71.25 (4.41)	Twice-weekly	60 min	4 weeks	RC with VHT/RC	GDS-30
Kim and Park (2018)	RCT	Korea	18E/18C	40–59	Twice-weekly	60 min	6 weeks	HT/ Take the pre-test and post-test only	SDS
Lin et al. (2022)	RCT	China	45E/45C	54.58 (4.36) / 53.98 (4.45)	Twice-weekly	30 min	12 weeks	FHT with RC/RC	SDS
Su et al. (2021)	RCT	China	58E/59C	57.01(10.65) / 54.73(10.59)	Twice-weekly	30 min	12 weeks	CHT with RC/RC	HAMD
Su et al. (2021)	RCT	China	58E/59C	57.01(10.65) / 54.73(10.59)	Twice-weekly	30 min	4 weeks	CHT with RC/RC	HAMD
Makizako et al. (2020)	RCT	Japan	26E/28C	73.1(5.6) / 73.0 (5.9)	A total of 20 treatments were performed in 6 months	60–90 min	6 months	HT/EC	GDS-15
Park et al. (2016)	Quasi-experiment	Korea	24E/26C	79.4(4.8)/ 84.5(4.7)	Twice-weekly	50 min	7.5 weeks	HT/NOI	GDSSF-K
Hyun et al. (2020b)	Quasi-experiment	Korea	10E/9C	58.78(9.56)/ 61.3(12.40)	Twice-weekly	90–120 min	4 weeks	HT/NOI	CES-D
Hoseinpoor Najjar et al. (2018)	Quasi-experiment	Iran	15E/15C	NA	Twice-weekly	120 min	5 weeks	HT/NOI	DASS44-D
Curzio et al. (2022)	RCT	Italy	6E/6C	14.86(1.92)	Twice-weekly	45 min	12 weeks	HT with RC/RC	CDI
Hyun et al. (2020a)	Quasi-experiment	Korea	13(Pre-and post-test study)	81.69(3.12)	Once-weekly	100 min	15 weeks	HT(Pre-and post-test study)	GDS-15
Hyun et al. (2020a)	Quasi-experiment	Korea	8(Pre-and post-test study)	81.88(4.26)	Twice-weekly	100 min	7.5 weeks	HT(Pre-and post-test study)	GDS-15
Kelley et al. (2017)	Quasi-experiment	America	8E/9C	31–35/26–30	At least once a week	60 min	6 weeks	HT/NOI	DASS-21-D
Lee et al. (2020)	Quasi-experiment	Korea	16(Pre-and post-test study)	37.81(3.45)	Once-weekly	90 min	6 weeks	HT(Pre-and post-test study)	K-BDI
Ruth et al. (2011)	Quasi-experiment	America	13E/13C	73.90(6.79)/ 74.3(6.40)	Twice-weekly	60–120 min	6 weeks	HT/AT	GDS-30
Baik et al. (2024)	RCT	Korea	29E/30C	41.55(4.14) / 43.73(4.14)	4 months (15 sessions)	About 180 min	4 months	HT/NOI	MHS:D
Chen and Ji (2015)	Quasi-experiment	China	10(Pre-and post-test study)	75.3 (9.55)	Once-weekly	90 min	5 weeks	HT(Pre-and post-test study)	GDS-SF-15
Chen and Ji (2015)	Quasi-experiment	China	10(Pre-and post-test study)	75.3 (9.55)	Once-weekly	90 min	10 weeks	HT(Pre-and post-test study)	GDS-SF-15
Cui et al. (2019)	RCT	China	40E/40C	48.7(9.3)/ 49.5(9.5)	Five times-weekly	45–60 min	4 weeks	HT with RC/RC	HAMD-24
Kam and Siu (2010)	RCT	China	10E/12C	45.3(10.38)/ 43.3(11.7)	10 times in a row	60 min	10 times in a row	HT with CT/CT	DASS21-D
Shi et al. (2023)	RCT	China	62E/62C	49.33(12.20)/ 47.28(12.15)	Five times-weekly	60–120 min	3 months	HT with RC/RC	SDS
Wei (2020)	RCT	China	41E/41C	61.26(5.91)/ 62.17(5.86)	Thrice-weekly	60 min	12 weeks	HT with RC/RC	HAMD-17
Wu et al. (2018)	RCT	China	67E/67C	35.15(10.49)/ 34.66(10.26)	Five times-weekly	60–120 min	8 weeks	HT with RC/RC	HAMD-24
Wu et al. (2018)	RCT	China	67E/67C	35.15(10.49)/ 34.66(10.26)	Five times-weekly	60–120 min	4 weeks	HT with RC/RC	HAMD-24
Xu et al. (2022)	RCT	China	30E/30C	60–90	Thrice-weekly	60 min	6 months	HT with RC/RC	HAMD
Xu et al. (2022)	RCT	China	30E/30C	60–90	Thrice-weekly	60 min	12 months	HT with RC/RC	HAMD
Yan et al. (2017)	RCT	China	35E/35C	57.5(9.2)/ 59.6(5.7)	Once a day	30 min	4 weeks	HT with RC/RC	BDI
Wen et al. (2020)	RCT	China	30E/30C	63.74(3.24)/ 64.10(3.56)	Twice a day	15 min	4 weeks	HTG with COPD /COPD	HAM-D
Wang et al. (2016)	RCT	China	80E/80C	60.23(13.12)/ 59.64(14.38)	Twice a day	60 min	4 weeks	HTG with RC/RC	HAMD-17
Hitter et al. (2019)	Quasi-experiment	Romania	8(Pre-and post-test study)	19–32	10 consecutive days (including 2 days off on weekends)	4 h	2 weeks	HT(Pre-and post-test study)	BDI
Rajoo et al. (2021)	Quasi-experiment	Malaysia	15(Pre-and post-test study)	26.2(4.14)	Once a day	20 min	1 weeks	HT(Pre-and post-test study)	DASS21-D
Kotozaki (2014)	RCT	Japan	22E/23C	46.53(8.40)	Once a day	Once a day(15 min) with Once-weekly(120 min)	16 weeks	HT/NOI	CES-D

E, experimental group; C, control group. HT, horticultural therapy program; VHT, virtual horticultural therapy; FHT, five sense stimulation horticultural therapy; CHT, chinese herbal horticultural therapy; HTG, rehabilitation garden was used for rehabilitation training; RC, routine care; EC, educational control group; NOI, not participate in any gardening intervention (not participate in horticultural therapy); CT, conventional sheltered workshop training; COPD, a stable COPD medication-assisted treatment plan; AT, Art Therapy.

**Table 2:** Risk of bias in quasi-experimental studies.

Include study	1	2	3	4	5	6	7	8	9
Park et al. (2016)	Yes	No	Yes	Yes	Yes	Yes	Yes	Yes	Yes
Hyun et al. (2020a)	Yes	Yes	Yes	Yes	Yes	Yes	Yes	Yes	Yes
Hoseinpoor Najjar et al. (2018)	Yes	Yes	Yes	Yes	Yes	Yes	Yes	Yes	Yes
Ruth et al. (2011)	Yes	Yes	No	Yes	Yes	Yes	Yes	Yes	Yes
Kelley et al. (2017)	Yes	Yes	Yes	Yes	Yes	Yes	Yes	Yes	Yes
Hitter et al. (2019)	Yes	Yes	Yes	No	Yes	Yes	Yes	Yes	Yes
Rajoo et al. (2021)	Yes	Yes	Yes	No	Yes	Yes	Yes	Yes	Yes
Lee et al. (2020)	Yes	Yes	Yes	No	Yes	Yes	Yes	Yes	Yes
Hyun et al. (2020b)	Yes	Yes	Yes	No	Yes	Yes	Yes	Yes	Yes
Chen and Ji (2015)	Yes	Yes	Yes	No	Yes	Yes	Yes	Yes	Yes

1. Is it clear in the study what is the “cause” and what is the “effect” (i.e., there is no confusion about which variable comes first)?2. Were the participants included in any comparisons similar?3. Were the participants included in any comparisons receiving similar treatment/care, other than the exposure or intervention of interest?4. Was there a control group?5. Were there multiple measurements of the outcome both pre and post the intervention/exposure?6. Was follow-up complete and, if not, were differences between groups in terms of their follow-up adequately described and analyzed?7. Were the outcomes of participants included in any comparisons measured in the same way?8. Were outcomes measured in a reliable way?9. Was appropriate statistical analysis used?

### 3.2 Study characteristics

Among the 29 articles (encompassing 33 studies), publication years spanned 2010–2024, including 21 RCTs (19 articles) and 12 quasi-experimental studies (10 articles). Regarding intervention frequency, 19 studies implemented HT sessions less than 3 times weekly, while 14 studies conducted sessions 3 or more times weekly. Session duration exceeded 60 min in 16 studies and was ≤60 min in 17 studies. Intervention duration was categorized as follows: ≤4 weeks (11 studies), 5–8 weeks (11 studies), and ≥9 weeks (11 studies). Participant age (Ages means) distribution was as follows: >60 years (14 studies), 58–61 years (2 studies), 18–60 years (15 studies), 0–18 years (1 study), and the data unreported in one study. Most trials utilized hybrid indoor-outdoor settings combining planting and fabrication activities, except one that employed virtual reality (VR). Geographically, the studies originated from China (17 studies), Korea (7 studies), Japan (2 studies), America (2 studies), Poland (1 study), Italy (1 study), Romania (1 study), Malaysia (1 study), and Iran (1 study).

### 3.3 Horticultural therapy’s effectiveness on depressive symptoms

**Figure 4:**
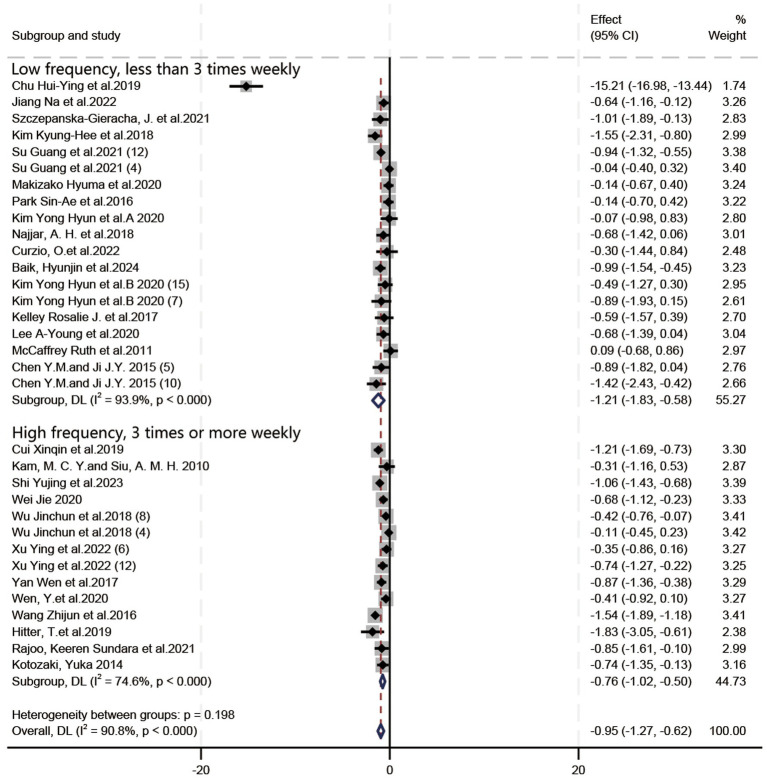
Forest plot: the impact of the frequency of horticultural therapy on the therapeutic efficacy for depressive symptoms.

**Figure 5:**
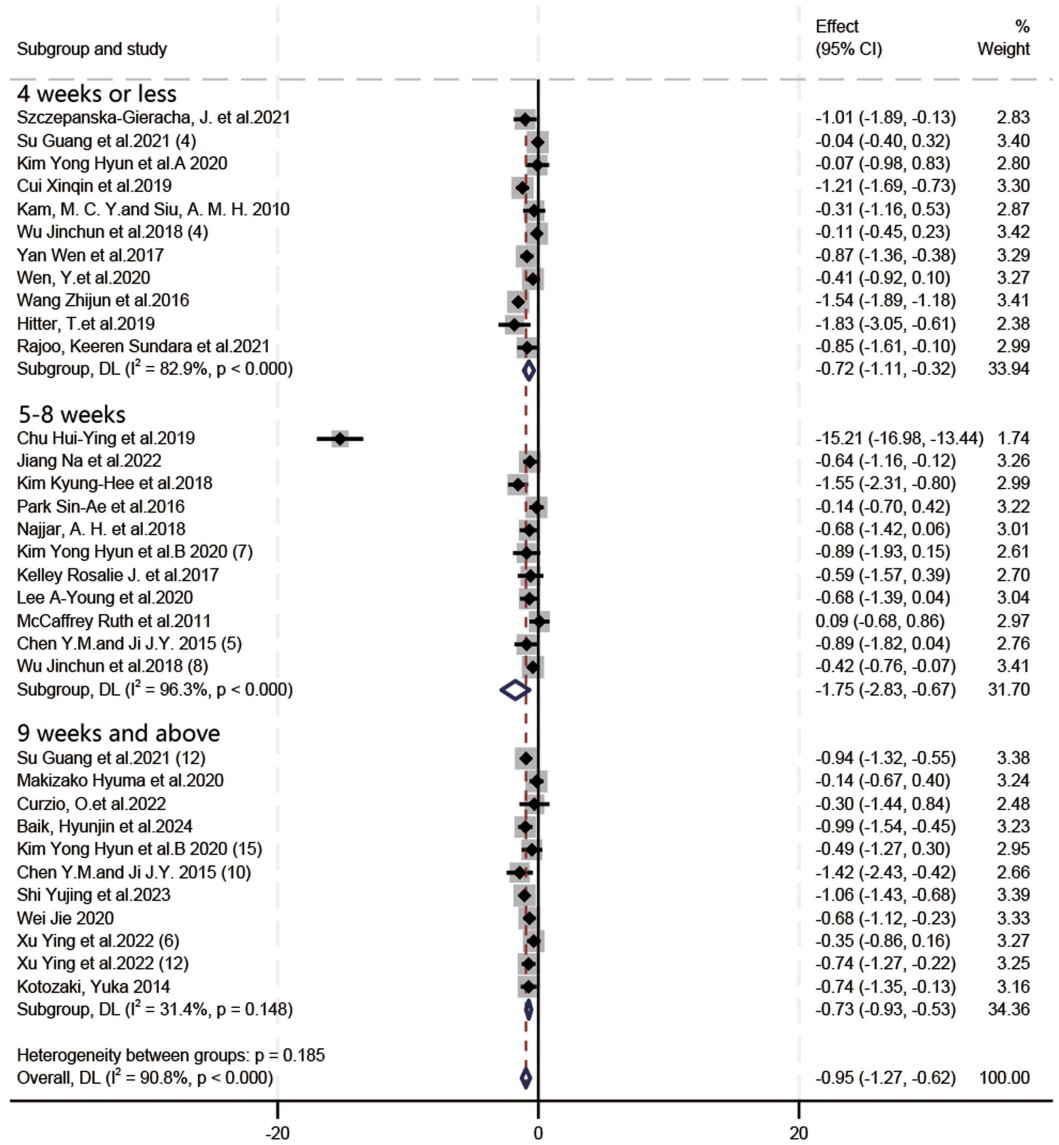
Forest plot: the impact of the intervention duration in horticultural therapy on the therapeutic efficacy for depressive symptoms.

**Figure 6:**
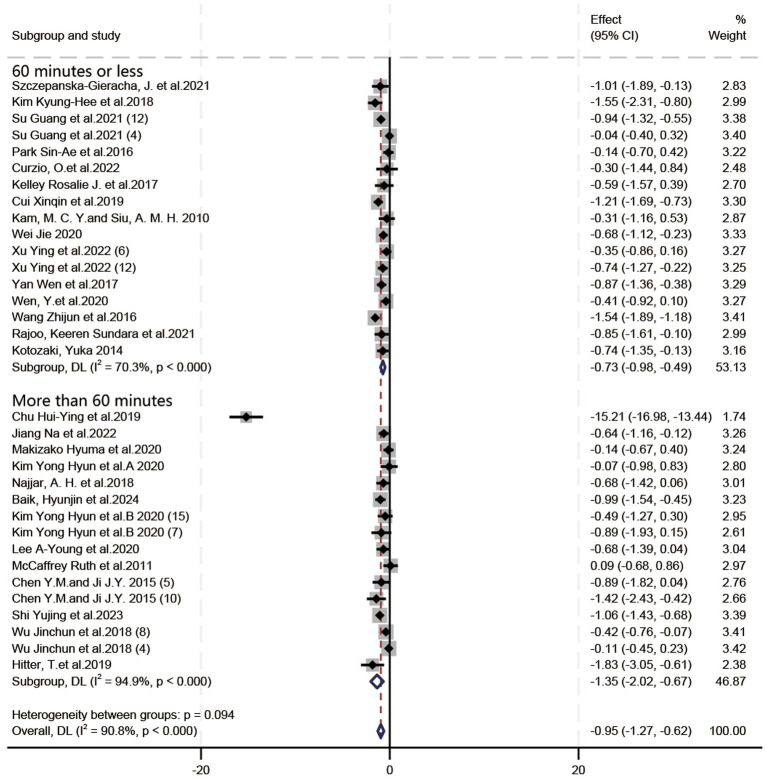
Forest plot: the impact of the session duration in horticultural therapy on the therapeutic efficacy for depressive symptoms.

In all, 21 RCTs (19 articles) and 12 quasi-experimental studies (10 articles) were included. Owing to the high level of heterogeneity, a random effects model and SMD were used for analysis. Based on the forest plots (Figures 4–6), HT exhibited a positive effect on depressive symptoms treatment, with a noteworthy effect size (SMD = −0.95, 95%CI = [−1.27, −0.62], *p* < 0.00001). Substantial heterogeneity was observed during the synthesis process of these studies (*I^2^* = 90.8%). By means of a case-by-case exclusion method, we found that the sensitivity was significantly reduced by removing one study (Chu et al., 2019), with the *I*^2^ decreasing to 65.2% (SMD = −0.68, 95%CI = [−0.85, −0.51], *p* < 0.00001). Nevertheless, this research suggested the considerable impact of HT on depression in elderly individuals and held certain reference value (Xu et al., 2023). Since the *I^2^* values for the included literature exceeded 50% during forest plot generation, further exploration into heterogeneity was performed through meta-regression analysis using various groups including frequency (Group 1), session duration (Group 2), intervention duration (Group 3), age (ages means) (Group 4), type of experiment (Group 5), type of scale (Group 6), and country/region (Group 7); however, *p* > 0.05 indicated that no source for heterogeneity had been identified yet.

### 3.4 Subgroup analysis of temporal characteristics

#### 3.4.1 Intervention frequency

In terms of the influence of intervention frequency on the therapeutic efficacy of HT in depressive symptoms (Figure 4), the included studies were classified into low-frequency (less than 3 times weekly) and high-frequency (3 or more times weekly) groups based on their characteristics. SMD was employed due to inconsistent criteria, and a random-effects model was utilized to deal with substantial heterogeneity (*I*^2^ = 90.8%). Figure 4 illustrates that low-frequency HT yielded a therapeutic effect (SMD = −1.21, 95%CI = [−1.83, −0.58], *p* = 0.0002), while high-frequency HT exhibited an (SMD = −0.76, 95%CI = [−1.02, −0.50], *p* < 0.00001). In the treatment of depressive symptoms, the effect of low-frequency HT was greater than that of high-frequency HT.

When one study was excluded (Chu et al., 2019), the overall heterogeneity was significantly reduced (*I*^2^ = 65.2%). At this point, the direction of subgroup analysis results was reversed: the treatment effect in the high-frequency group (SMD = −0.76, 95%CI = [−1.02, −0.50], *p* < 0.00001) was superior to that in the low-frequency group (SMD = −0.60, 95%CI = [−0.83, −0.37], *p* < 0.00001).

#### 3.4.2 Intervention duration

In terms of the impact of HT intervention duration on the effectiveness of HT in depressive symptoms (Figure 5), SMD was employed due to inconsistent assessment criteria. Given the substantial heterogeneity (*I*^2^ = 90.8%), a random-effects model was utilized. As shown in Figure 5, HT exhibited the following effect sizes: (SMD = −0.72, 95%CI = [−1.11, −0.32], *p* = 0.0004) for interventions lasting ≤4 weeks; (SMD = −1.75, 95%CI = [−2.83, −0.67], *p* = 0.001) for interventions lasting 5–8 weeks; and (SMD = −0.73, 95%CI = [−0.93, −0.53], *p* < 0.00001) for interventions lasting ≥9 weeks. Therefore, HT interventions lasting 5–8 weeks are likely to be more effective.

When one study was excluded (Chu et al., 2019), the overall heterogeneity was significantly reduced (*I*^2^ = 65.2%). At this point, the direction of subgroup analysis results was reversed: the treatment effect of 5–8 weeks of HT (SMD = −0.58, 95%CI = [−0.84, −0.32], *p* < 0.0001) was less effective than that of interventions lasting ≤4 weeks (SMD = −0.72, 95%CI = [−0.11, −0.32], *p* = 0.0004) or ≥9 weeks (SMD = −0.73, 95%CI = [−0.93, −0.53], *p* < 0.00001).

#### 3.4.3 Session duration

In terms of the influence of session duration on the effectiveness of HT in depressive symptoms (Figure 6), SMD was utilized due to inconsistent assessment criteria. Given the substantial heterogeneity (*I*^2^ = 90.8%), a random-effects model was employed. As depicted in Figure 6, HT exhibited a larger therapeutic effect in activities lasting >60 min (SMD = −1.35, 95%CI = [−2.02, −0.67], *p* < 0.0001), compared to those lasting ≤60 min (SMD = −0.73, 95%CI = [−0.98, −0.49], *p* < 0.00001). Therefore, HT sessions lasting >60 min demonstrated the most favorable therapeutic effects.

When one study was excluded (Chu et al., 2019), the overall heterogeneity was significantly reduced (*I*^2^ = 65.2%). At this point, the direction of subgroup analysis results was reversed: the treatment effect of HT lasting ≤60 min (SMD = −0.73, 95%CI = [−0.98, −0.49], *p* < 0.00001) demonstrated a larger effect size than HT lasting >60 min (SMD = −0.60, 95%CI = [−0.84, −0.37], *p* < 0.00001).

#### 3.4.4 Combination of intervention frequency and duration

**Figure 7:**
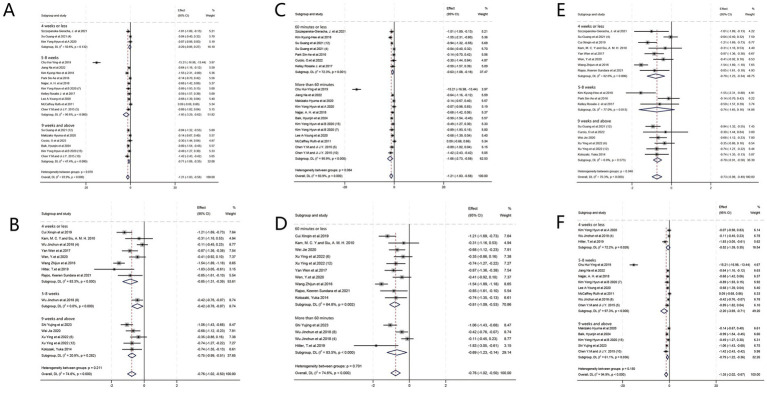
Forest plot: combinatory analysis. **(A)** Relationship between frequency and duration of intervention (low frequency). **(B)** Relationship between frequency and duration of intervention (high frequency). **(C)** Relationship between frequency and session duration (low frequency). **(D)** Relationship between frequency and session duration (high frequency). **(E)** Relationship between duration of intervention and session duration (60 min or less). **(F)** Relationship between duration of intervention and session duration (more than 60 min).

Low-frequency HT for a duration of 5–8 weeks exhibited the most significant therapeutic effect (SMD = −1.95, 95%CI = [−3.29, −0.62], *p* = 0.004) (Figures 7A,B).

When the study Chu et al. (2019) was excluded, the heterogeneity of the forest plot (Figure 7A) was significantly reduced (*I^2^* = 50.6%). At this point, the results of the subgroup analysis changed: HT treatment with a high frequency of 4 weeks or less was the most effective (SMD = −0.85, 95%CI = [−1.31, −0.39], *p* = 0.0003).

#### 3.4.5 Combination of intervention frequency and session duration

Regarding depressive symptoms treatment, long-session duration HT (>60 min per session) with low-frequency interventions (<3 times weekly) exhibited the most substantial therapeutic effect (SMD = −1.66, 95%CI = [−2.73, −0.58], *p* = 0.003) (Figures 7C,D).

When the study (Chu et al., 2019) was excluded, forest plot (Figure 7C) heterogeneity was significantly reduced (*I*^2^ = 50.6%). At this point, the results of the subgroup analysis changed: HT with high-frequency sessions of 60 min or less was the most effective treatment (SMD = −0.81, 95%CI = [−1.09, −0.53], *p* < 0.00001).

#### 3.4.6 Combination of intervention duration and session duration

Regarding the relationship between the intervention duration of HT and session duration in treating depressive symptoms, HT exhibited superior therapeutic efficacy for a span of 5–8 weeks with long-session duration (>60 min) (SMD = −2.20, 95%CI = [−3.69, −0.71], *p* = 0.004) (Figures 7E,F).

When the study by Chu et al. (2019) was excluded, forest plot (Figure 7F) heterogeneity was significantly reduced (*I*^2^ = 54.9%). At this point, the results of the subgroup analysis changed: HT therapy lasting 60 min or less for 4 weeks or less (SMD = −0.79, 95%CI = [−1.23, −0.34], *p* = 0.0005) and HT lasting more than 60 min for 9 weeks and above were more effective (SMD = −0.79, 95%CI = [−1.22, −0.36], *p* = 0.0003).

#### 3.4.7 Comparison of effect sizes of different combinations of programs

On comparing the results of the three combinations of intervention frequency and duration, intervention frequency and session duration, and intervention duration and session duration, we found that 5–8 weeks, more than 60 min, and low frequency were recurrently validated against each other, and thus the combination of the three may produce better results (Table 3).

**Table 3:** Statistical table of effect sizes of time-factor combination programs.

Combining scheme	Std. mean difference [95%CI]	*I*^2^	*p*-value
Low frequency × 4 weeks or less	−0.29 [−0.86, 0.27]	50.6%	0.31
Low frequency × 5–8 weeks	−1.95 [−3.29, −0.62]	96.6%	0.004
Low frequency × 9 weeks and above	−0.71 [−1.08, −0.35]	47.4%	0.0001
High frequency × 4 weeks or less	−0.85 [−1.31, −0.39]	83.3%	0.0003
High frequency × 5–8 weeks	−0.42 [−0.76, −0.07]	0.0%	0.02
High frequency × 9 weeks and above	−0.75 [−0.99, −0.51]	20.9%	P < 0.00001
Low frequency × 60 min or less	−0.63 [−1.08, −0.18]	72.3%	0.006
Low frequency × more than 60 min	−1.66 [−2.73, −0.58]	95.9%	0.003
High frequency × 60 min or less	−0.81 [−1.09, −0.53]	64.8%	*p* < 0.00001
High frequency × more than 60 min	−0.69 [−1.23, −0.14]	83.5%	0.01
60 min or less × 4 weeks or less	−0.79 [−1.23, −0.34]	82.6%	0.0005
60 min or less × 5–8 weeks	−0.74 [−1.65, 0.16]	77.0%	0.11
60 min or less × 9 weeks and above	−0.70 [−0.91, −0.50]	0.0%	*p* < 0.00001
More than 60 min × 4 weeks or less	−0.52 [−1.39, 0.35]	72.2%	0.24
More than 60 min × 5–8 weeks	−2.20 [−3.69, −0.71]	97.3%	0.004
More than 60 min × 9 weeks and above	−0.79 [−1.22, −0.36]	61.1%	0.0003

When one study was removed (Chu et al., 2019), the subgroup heterogeneity involving that study was significantly reduced and the results of the subgroup analysis changed.

### 3.5 Publication bias

**Figure 8:**
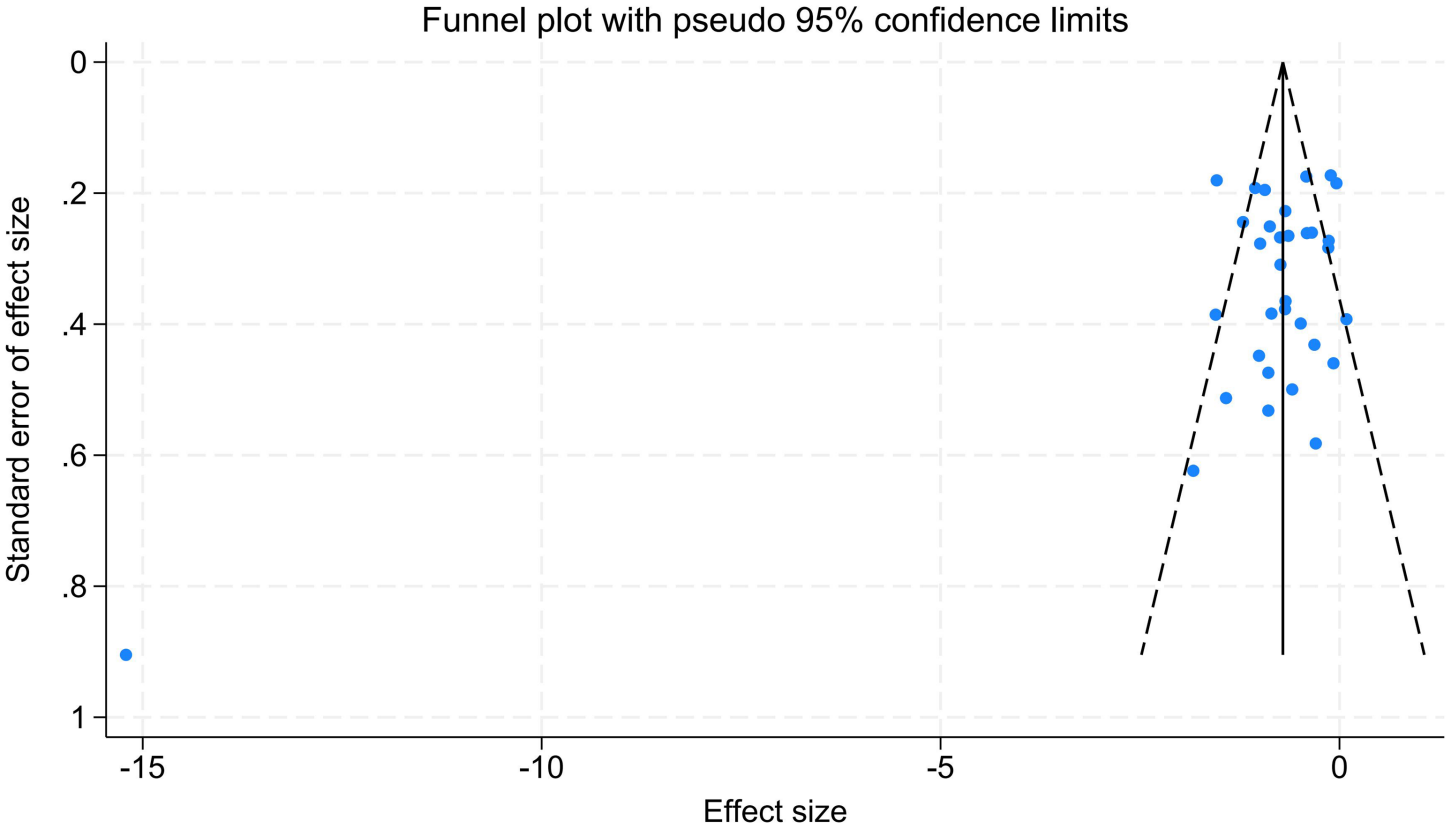
Funnel plot.

According to the funnel plot (Figure 8), the studies included in this study were predominantly concentrated in the central and upper regions, with only one study located at the lower end. The overall symmetrical distribution indicates a lack of significant deviation. Additionally, Egger’s test (*p* = 0.060; *p* > 0.05) did not yield substantial evidence of bias.

## 4 Discussion

### 4.1 Influence of intervention frequency

The results suggest that the frequency of intervention in HT may influence its therapeutic effect. Specifically, lower intervention frequencies (<3 times weekly) might lead to better outcomes in alleviating depressive symptoms. In our meta-analysis, the results of low-frequency HT interventions were more favorable than those of high-frequency interventions. From an implementation perspective, determining the treatment frequency in HT requires balancing of natural biological rhythms and psychological recovery mechanisms (Shuhua et al., 2019). Low-frequency HT allows individuals to maintain autonomy in their interactions with plants, aligns with neuroendocrine regulation cycles, and enhances the sustainability of treatment effects. Designing low-frequency gardening activities not only alleviates the pressure associated with high-frequency interventions but also synchronizes with the natural growth cycles of plants, thereby stimulating participants’ expectations and sense of responsibility (Xu et al., 2023).

### 4.2 Influence of intervention duration

Research demonstrates that the intervention duration of HT significantly impacts therapeutic efficacy. Through systematic analysis of RCTs and quasi-experimental studies, interventions were categorized into three duration groups: ≤4 weeks, 5–8 weeks, and ≥9 weeks. Subgroup analyses indicated that interventions lasting 5–8 weeks produced the best therapeutic results, while programs ≤4 weeks or ≥9 weeks showed no significant difference in alleviating depressed symptoms, both showing lower efficacy than the 5–8 week duration. From an implementation perspective, a moderate duration (5–8 weeks) balances two challenges: programmatic pressures from long-duration interventions and discontinuity in short-duration interventions. This balance enhances feasibility by maintaining appropriate intervention density while allowing autonomous recovery. Shorter duration (≤4 weeks) often results in insufficient engagement and reduced effectiveness. Existing research supports that brief non-pharmacological treatments for depression are generally less effective (Xu et al., 2023). Second, the scientific validity of this duration is further supported by behavior change theory. Prochaska’s stages of change model emphasizes that healthy behaviors develop through a gradual process (Prochaska et al., 1992). The 5–8 week duration covers a critical transition duration: the first 2–4 weeks help participants build foundational skills and interests, and the last 2–4 weeks reinforce self-efficacy through visualization of outcomes (plants blooming and fruiting). For example, a meta-analysis on psychotherapy for postpartum depression revealed significant differences in EPDS scores between the intervention and control groups only when the psychotherapy lasted ≥8 weeks. However, beyond this duration, no significant differences were observed (Dong et al., 2013).

The duration and frequency of interventions are closely intertwined in the actual operation of HT. Longer intervention duration is typically associated with lower intervention frequencies, whereas shorter interventions tends to have higher frequencies. Our analysis of the combination of frequency and duration suggests that low-frequency treatment for 5–8 weeks is optimal, followed by high-frequency treatment for 4 weeks and less or 9 weeks and above, and low-frequency HT for 9 weeks and above also has some effect. The results of the study show that it takes some time cumulatively to achieve good results in HT for the treatment of depressive symptoms.

### 4.3 Influence of session duration

In terms of the session duration of HT, our study suggests that sessions lasting more than 60 min yield the most effective results. According to the Attention Curve Theory proposed by American psychologist Dr. Lucy Jo Palladino, a person’s attention can be outlined using an inverted U-shaped attention curve (Palladino, 2007). For HT, a certain amount of session time is needed to give participants a sense of engagement and immersion. Analysis of the results combining session duration and frequency of interventions showed that prolonged HT (more than 60 min) in conjunction with low-frequency interventions (less than 3 times weekly) showed the most significant therapeutic effects. Analysis combining session duration with duration showed that HT demonstrated superior therapeutic efficacy in a single session (more than 60 min) over a duration of 5–8 weeks. This finding is in contrast with the effect of intervention therapy, another non-drug treatment. In a study on exercise intervention in depression treatment (Liu et al., 2025), exercise interventions lasting <60 min were found to be superior to those lasting >60 min in improving cognitive function.

### 4.4 Combined influence of temporal characteristics

On analyzing the frequency, duration, and session duration of HT interventions, the following patterns emerged: Low-frequency HT interventions (less than 3 times weekly) over 5–8 weeks yield superior outcomes. Interventions lasting >60 min per session across 5–8 weeks yield superior outcomes. Low-frequency interventions (less than 3 times weekly) with sessions >60 min may optimize therapeutic effects. On comparing the three combinations (frequency-duration, frequency-session duration, and duration-session duration), we found that the parameters of 5–8 weeks, >60 min per session, and low frequency were mutually validated, indicating that their combined use may yield optimize outcomes. As a non-pharmacological treatment, HT efficacy can be influenced by multiple factors. Therefore, selecting appropriate duration, frequency, and session duration is critical for designing effective HT programs (Ascencio, 2019; Joubert et al., 2024). Combined analysis indicates that high-frequency interventions are unnecessary for achieving therapeutic goals, significantly enhancing program flexibility and accessibility. In conclusion, evidence suggests that ensuring session duration >60 min, combined with a duration of 5–8 weeks and low frequency (less than 3 times weekly), produces optimal results. HT programs for depressive symptoms should prioritize this regimen to maximize its potential as a psychologically supportive intervention.

### 4.5 Heterogeneity discussion

On excluding studies one-by-one, we found that one of the studies contributed a larger source of heterogeneity and its exclusion would have a significant effect on the SMD values (Chu et al., 2019). The analysis showed that this study was an RCT experiment, and the ROB 2 risk assessment met the criteria and the large sample size. Analysis of the results after excluding this study showed that the results were difficult to interpret and the conclusions lacked robustness. The study was also included in a meta-analysis of other horticultural therapies (Xu et al., 2023). In summary, this study did not exclude this study, suggesting that the current literature on HT lacks rigor and that relevant research literature is needed to confirm the article’s conclusions, highlighting the need for RCTs or quasi-experiment studies using standardized HT protocols.

### 4.6 Application

We found that low-frequency interventions (less than 3 times weekly) over 5–8 weeks, with each session lasting >60 min, can achieve optimal therapeutic outcomes, demonstrating that HT is characterized by flexibility and continuity. HT is not limited to clinical settings such as hospitals and rehabilitation centers but can also be implemented in communities or campuses. Sessions lasting >60 min may enhance efficacy, suggesting that HT requires a certain degree of immersion to achieve optimal effects. This finding addresses concerns that prolonged HT interventions might elicit negative reactions in elderly individuals or psychologically vulnerable populations. In summary, HT has proven highly effective in alleviating depressive symptoms and holds significant potential for broader implementation.

## 5 Limitations

Multiple depression scales were used in this study. Even though meta-regression analyses were conducted for this influence and random effects models and SMD were used to reduce the effects of high heterogeneity, this study has some limitations. In terms of the study’s temporal characteristics, HT experiments exceeding 9 weeks and lasting over 60 min were not analyzed further. With regard to the environmental setting, no further distinction was made between specific intervention settings in this study. In terms of publication bias, a large proportion of studies were conducted in Asia, and this could introduce bias in the results of meta-analysis. Egger’s test for publication bias showed a *p*-value of 0.060, which was not statistically significant (*p* > 0.05), but it was close to the critical threshold, indicating the presence of some risk. Demographic characteristics such as gender and age were not further disaggregated when exploring the influence of the temporal characteristics due to limitations in the number of HT studies. Additionally, only Chinese and English journals were considered in this study, potentially overlooking articles related to HT published in other languages. Although subgroup analyses identified differences in the temporal characteristics in the treatment of depressive symptoms with HT, meta-regression analyses indicated that this was not a major source of heterogeneity, suggesting that differences between subgroups may be limited and that more relevant research is needed to justify the article’s conclusions.

When it comes to evaluating the quality of the literature, as illustrated in Figure 2, many articles failed to explicitly state whether blinding was implemented. As shown in Table 2, many quasi-experimental studies have certain risks. A more detailed analysis of participants’ baseline information and post-experiment follow-up should be conducted in subsequent HT trials.

## 6 Conclusion

Research reveals the role of time effects in HT for the treatment of depressive symptoms. The temporal characteristics (intervention frequency, intervention duration, session duration) have an important effect on the therapeutic effect of HT. In summary, when developing an HT program for the treatment of depressive symptoms, it is recommended that activities be performed less than 3 times weekly for 5–8 weeks, and that each session should be more than 60 min. This finding demonstrates the high flexibility and replicability of HT programs, which facilitates the extension of HT to a wider and more diverse population. It is worth noting that a high degree of heterogeneity was found during the meta-analysis. Therefore, more relevant RCTs or quasi-experimental studies are needed in the future to validate the effect of the temporal characteristics on HT for the treatment of depressive symptoms. The present study provides evidence in the form of a meta-analysis on the implementation of HT programs focused on intervening in depressive symptoms, which complements the lack of findings on the temporal dimension of current research in this field and contributes to the promotion of the use of HT in the treatment of depressive symptoms.

## Data Availability

The original contributions presented in the study are included in the article/supplementary material, further inquiries can be directed to the corresponding author.

## References

[ref1] Ascencio J. (2019). Horticultural therapy as an intervention for schizophrenia: a review. Altern. Complement. Ther. 25, 194–200. doi: 10.1089/act.2019.29231.jas

[ref2] Baik H. Choi S. An M. Jin H. Kang I. Yoon W. . (2024). Effect of therapeutic gardening program in urban gardens on the mental health of children and their caregivers with atopic dermatitis. Healthcare 12:919. doi: 10.3390/healthcare12090919, PMID: 38727476 PMC11083003

[ref3] Barker T. H. Habibi N. Aromataris E. Stone J. C. Leonardi-Bee J. Sears K. . (2024). The revised JBI critical appraisal tool for the assessment of risk of bias for quasi-experimental studies. JBI Evid. Synth. 22, 378–388. doi: 10.11124/JBIES-23-00268, PMID: 38287725

[ref4] Briggs R. Morris P. G. Rees K. (2023). The effectiveness of group-based gardening interventions for improving wellbeing and reducing symptoms of mental ill-health in adults: a systematic review and meta-analysis. J. Ment. Health 32, 787–804. doi: 10.1080/09638237.2022.2118687, PMID: 36151719

[ref5] Bruijniks S. J. E. Lemmens L. Hollon S. D. Peeters F. Cuijpers P. Arntz A. . (2020). The effects of once- versus twice-weekly sessions on psychotherapy outcomes in depressed patients. Br. J. Psychiatry 216, 222–230. doi: 10.1192/bjp.2019.265, PMID: 32029012

[ref6] Chan H. Y. Ho R. C. M. Mahendran R. Ng K. S. Tam W. W. S. Rawtaer I. . (2017). Effects of horticultural therapy on elderly' health: protocol of a randomized controlled trial. BMC Geriatr. 17:192. doi: 10.1186/s12877-017-0588-z, PMID: 28851276 PMC5576101

[ref7] Chen Y. M. Ji J. Y. (2015). Effects of horticultural therapy on psychosocial health in older nursing home residents a preliminary study. J. Nurs. Res. 23, 167–171. doi: 10.1097/jnr.0000000000000063, PMID: 25534575

[ref8] Chu H. Y. Chen M. F. Tsai C. C. Chan H. S. Wu T. L. (2019). Efficacy of a horticultural activity program for reducing depression and loneliness in older residents of nursing homes in Taiwan. Geriatr. Nurs. 40, 386–391. doi: 10.1016/j.gerinurse.2018.12.012, PMID: 30792050

[ref9] Cipriani J. Benz A. Holmgren A. Kinter D. McGarry J. Rufino G. (2017). A systematic review of the effects of horticultural therapy on persons with mental health conditions. Occup. Ther. Ment. Health 33, 47–69. doi: 10.1080/0164212x.2016.1231602

[ref10] Cui X. Xu W. Lyu X. (2019). Effects of horticultural therapy on rehabilitation of hospitalized patients with depression. Chin. J. Pract. Nurs. 35, 1011–1014. doi: 10.3760/cma.j.issn.1672-7088.2019.13.012

[ref11] Cumpston M. Li T. J. Page M. J. Chandler J. Welch V. A. Higgins J. P. T. . (2019). Updated guidance for trusted systematic reviews: a new edition of the Cochrane handbook for systematic reviews of interventions. Cochrane Database Syst. Rev. 10:ED000142. doi: 10.1002/14651858.ED000142, PMID: 31643080 PMC10284251

[ref12] Curzio O. Billeci L. Belmonti V. Colantonio S. Cotrozzi L. De Pasquale C. F. . (2022). Horticultural therapy may reduce psychological and physiological stress in adolescents with anorexia nervosa: a pilot study. Nutrients 14:5198. doi: 10.3390/nu14245198, PMID: 36558357 PMC9786778

[ref13] Dong Y. Mao X. Liu Y. (2013). Effectiveness of psychotherapy in the management of postpartum depression: a meta-analysis. Prog. Obstet. Gynecol. 22, 377–381. doi: 10.13283/j.cnki.xdfckjz.2013.05.001

[ref14] Farah W. H. Alsawas M. Mainou M. Alahdab F. Farah M. H. Ahmed A. T. . (2016). Non-pharmacological treatment of depression: a systematic review and evidence map. Evid. Based Med. 21, 214–221. doi: 10.1136/ebmed-2016-110522, PMID: 27836921

[ref15] He M. Hu Y. Wen Y. Wang X. Wei Y. W. Sheng G. H. . (2024). The impacts of Forest therapy on the physical and mental health of college students: a review. Forests 15:682. doi: 10.3390/f15040682

[ref16] Higgins J. P. T. Altman D. G. Gotzsche P. C. Jüni P. Moher D. Oxman A. D. . (2011). The Cochrane Collaboration's tool for assessing risk of bias in randomised trials. Br. Med. J. 343:d5928. doi: 10.1136/bmj.d5928, PMID: 22008217 PMC3196245

[ref17] Hitter T. Kállay É. Olar L. E. Ştefan R. Buta E. Chiorean S. . (2019). The effect of therapeutic horticulture activities on people in depression and kynurenine pathways. Not. Bot. Horti Agrobot. Cluj Napoca 47, 804–812. doi: 10.15835/nbha47311544

[ref18] Hoseinpoor Najjar A. Foroozandeh E. Asadi Gharneh H. A. (2018). Horticulture therapy effects on memory and psychological symptoms of depressed male outpatients. Iran. Rehabil. J. 16, 147–154. doi: 10.32598/irj.16.2.147

[ref19] Hu Y. H. Xue H. Ni X. Y. Guo Z. Fan L. J. Du W. (2024). Association between duration of antidepressant treatment for major depressive disorder and relapse rate after discontinuation: a meta-analysis. Psychiatry Res. 337:115926. doi: 10.1016/j.psychres.2024.115926, PMID: 38733930

[ref20] Hyun K. Y. Jo H.-S. Soo P. C. Kang K. Lee E. S. Jo S. H. . (2020a). Comparing the effectiveness of the frequency and duration of the horticultural therapy program on elderly women with mild cognitive impairment and mild dementia. J. People Plants Environ. 23, 35–46. doi: 10.11628/ksppe.2020.23.1.35

[ref21] Hyun K. Y. Soo P. C. Bae H.-O. Lim E. J. Kang K. H. Lee E. S. . (2020b). Horticultural therapy programs enhancing quality of life and reducing depression and burden for caregivers of elderly with dementia. J. People Plants Environ. 23, 305–320. doi: 10.11628/ksppe.2020.23.3.305

[ref22] Jiang N. Hu H. Xie L. Qiu Y. (2022). Intervention study of horticultural therapy on depression in the elderly with mild to moderate cognitive disease. J. Yueyang Vocat. Tech. College 37, 74–79. doi: 10.13947/j.cnki.yyzyxb.2022.04.018

[ref23] Joubert A. Jankowski-Cherrier B. Rossi A. Teyssier L. Suraud V. Presle E. . (2024). Impact of horticultural therapy on patients admitted to psychiatric wards, a randomised, controlled and open trial. Sci. Rep. 14:14378. doi: 10.1038/s41598-024-65168-0, PMID: 38909093 PMC11193794

[ref24] Kam M. C. Y. Siu A. M. H. (2010). Evaluation of a horticultural activity programme for persons with psychiatric illness. Hong Kong J. Occup. Ther. 20, 80–86. doi: 10.1016/S1569-1861(11)70007-9

[ref25] Kaplan R. Kaplan S. (1989). The experience of nature: A psychological perspective. Cambridge University Press.

[ref26] Kelley R. J. Waliczek T. M. Le Duc F. A. (2017). The effects of greenhouse activities on psychological stress, depression, and anxiety among university students who served in the US armed forces. HortScience 52, 1834–1839. doi: 10.21273/HORTSCI12372-17

[ref27] Kim K. H. Park S. A. (2018). Horticultural therapy program for middle-aged women's depression, anxiety, and self-identify. Complement. Ther. Med. 39, 154–159. doi: 10.1016/j.ctim.2018.06.008, PMID: 30012387

[ref28] Kotozaki Y. (2014). Horticultural therapy as a measure for recovery support of regional community in the disaster area: a preliminary experiment for forty five women who living certain region in the coastal area of Miyagi prefecture. Int. J. Emerg. Ment. Health 16, 284–287. Available at: https://pubmed.ncbi.nlm.nih.gov/25585479/, PMID: 25585479

[ref30] Lee Y. W. Chen T. T. Hsu C. W. Chen M. D. Lin P. Y. Huang Y. C. . (2024). Efficacy of horticultural therapy on positive, negative, and affective symptoms in individuals with schizophrenia: a systematic review and Meta-analysis of randomized controlled trials. Healthcare 12:2104. doi: 10.3390/healthcare12212104, PMID: 39517317 PMC11545822

[ref31] Lee A. Y. Kim S. O. Gim G. M. Kim D. S. Park S. A. (2020). Care farming program for family health: a pilot study with mothers and children. Int. J. Environ. Res. Public Health 17:27. doi: 10.3390/ijerph17010027, PMID: 31861441 PMC6981719

[ref32] Lin L. Chen F. Wu C. (2022). Application of five-sense stimulating horticulture therapy in patients with maintenance hemodialysis. Chin. Clin. Nurs. 14, 339–342. doi: 10.3969/j.issn.1674-3768.2022.06.003

[ref33] Liu Y. Zhao G. R. Guo J. Qu H. Y. Kong L. L. Yue W. H. (2025). The efficacy of exercise interventions on depressive symptoms and cognitive function in adults with depression: An umbrella review. J. Affect. Disord. 368, 779–788. doi: 10.1016/j.jad.2024.09.074, PMID: 39278470

[ref34] Lu S. Liu J. J. Xu M. J. Xu F. (2023). Horticultural therapy for stress reduction: a systematic review and meta-analysis. Front. Psychol. 14:1086121. doi: 10.3389/fpsyg.2023.1086121, PMID: 37564307 PMC10411738

[ref35] Makizako H. Tsutsumimoto K. Doi T. Makino K. Nakakubo S. Liu-Ambrose T. . (2020). Exercise and horticultural programs for older adults with depressive symptoms and memory problems: a randomized controlled trial. J. Clin. Med. 9:99. doi: 10.3390/jcm9010099, PMID: 31906021 PMC7019282

[ref36] Meore A. Ganesh N. Sun S. N. Singer A. Byma L. Lorenzetti B. . (2024). Pilot study of telehealth delivery of horticultural therapy (TeleHT) as an acceptable intervention and in reducing suicide risk factors in veterans. Complement. Ther. Med. 85:103075. doi: 10.1016/j.ctim.2024.103075, PMID: 39147286 PMC12168245

[ref37] Moher D. Liberati A. Tetzlaff J. Altman D. G. Grp P. (2009). Preferred reporting items for systematic reviews and Meta-analyses: the PRISMA statement (reprinted from annals of internal medicine). Phys. Ther. 89, 873–880. doi: 10.1093/ptj/89.9.873, PMID: 19723669

[ref38] Page M. J. McKenzie J. E. Bossuyt P. M. Boutron I. Hoffmann T. C. Mulrow C. D. . (2021a). The PRISMA 2020 statement: an updated guideline for reporting systematic reviews. Br. Med. J. 372:n71. doi: 10.1136/bmj.n71, PMID: 33782057 PMC8005924

[ref39] Page M. J. Moher D. Bossuyt P. M. Boutron I. Hoffmann T. C. Mulrow C. D. . (2021b). PRISMA 2020 explanation and elaboration: updated guidance and exemplars for reporting systematic reviews. Br. Med. J. 372:n160. doi: 10.1136/bmj.n160, PMID: 33781993 PMC8005925

[ref40] Palladino L. J. (2007). Find your focus zone. New York, NY: Free Press.

[ref41] Park S. A. Lee A. Y. Son K. C. Lee W. L. Kim D. S. (2016). Gardening intervention for physical and psychological health benefits in elderly women at community centers. Horttechnology 26, 474–483. doi: 10.21273/horttech.26.4.474

[ref42] Peters M. D. J. Marnie C. Tricco A. C. Pollock D. Munn Z. Alexander L. . (2021). Updated methodological guidance for the conduct of scoping reviews. JBI Evid. Implement. 19, 3–10. doi: 10.1097/XEB.0000000000000277, PMID: 33570328

[ref43] Prochaska J. O. DiClemente C. C. Norcross J. C. (1992). In search of how people change. Applications to addictive behaviors. Am. Psychol. 47, 1102–1114. doi: 10.1037/0003-066X.47.9.1102, PMID: 1329589

[ref44] Rajoo K. S. Karam D. S. Abdu A. Rosli Z. Gerusu G. J. (2021). Addressing psychosocial issues caused by the COVID-19 lockdown: can urban greeneries help? Urban For. Urban Green. 65:127340. doi: 10.1016/j.ufug.2021.127340, PMID: 34512230 PMC8423708

[ref45] Ruth M. Patricia L. Thomas G. Reiko N. (2011). Garden walking and art therapy for depression in older adults: a pilot study. Res. Gerontol. Nurs. 4, 237–242. doi: 10.3928/19404921-20110201-01 21323299

[ref46] Seok J. W. Kim J. I. (2024). The efficacy of eye movement desensitization and reprocessing treatment for depression: a Meta-analysis and Meta-regression of randomized controlled trials. J. Clin. Med. 13:5633. doi: 10.3390/jcm13185633, PMID: 39337119 PMC11433385

[ref47] Shi Y. Li Y. Liu X. Xu S. (2023). Effect of horticultural therapy on cancer pain and negative emotion in patients with gynecological malignant tumor. Chin. Gen. Pract. Nurs. 21, 2959–2962. doi: 10.12104/j.issn.1674-4748.2023.21.020

[ref48] Shuhua L. Yanan Y. Chang L. Ning K. (2019). The effect and mechanism of GreenSpace on human physical and mental health:a proposal of green medicine. Chin. Landscape Archit. 35, 5–11. doi: 10.19775/j.cla.2019.06.0005

[ref49] Sorensen A. Jorgensen K. J. Munkholm K. (2022). Clinical practice guideline recommendations on tapering and discontinuing antidepressants for depression: a systematic review. Ther. Adv. Psychopharmacol. 12:20451253211067656. doi: 10.1177/20451253211067656, PMID: 35173954 PMC8841913

[ref50] Su G. Li J. Yan G. Liao X. Cai X. Zhou P. . (2021). Clinical effect of horticultural therapy of aromatic herbal medicine on post-stroke depression. Chin. Nurs. Res. 35, 4461–4464. doi: 10.12102/j.issn.1009-6493.2021.24.023

[ref51] Szczepanska-Gieracha J. Cieslik B. Serweta A. Klajs K. (2021). Virtual therapeutic garden: a promising method supporting the treatment of depressive symptoms in late-life: a randomized pilot study. J. Clin. Med. 10:1942. doi: 10.3390/jcm10091942, PMID: 34062721 PMC8125254

[ref52] Wang Z. Huang W. Yan W. Liang W. (2016). Effects of rehabilitation garden on hemiplegic stroke patients. J. Pract. Med. 32, 2091–2094. doi: 10.3969/j.issn.1006-5725.2016.13.005

[ref53] Wang M. Qian Y. Yu X. Xing Y. (2024). Effectiveness of horticultural therapy in older patients with dementia: a Meta-analysis systemic review. J. Clin. Nurs. 33, 4543–4553. doi: 10.1111/jocn.17444, PMID: 39275900

[ref54] Wei J. (2020). Effect of horticultural therapy on glucose and lipid metabolism,physical dysfunction and psychological and mental state in patients with cerebral infarction. Chin. J. Health Psychol. 28, 25–29. doi: 10.13342/j.cnki.cjhp.2020.01.007

[ref55] Wen Y. Lian L. Xun Z. Jiaying M. Cui S. He W. . (2020). Effect of a rehabilitation garden on rehabilitation efficacy in elderly patients with chronic obstructive pulmonary disease. Pak. J. Zool. 52, 2393–2396. doi: 10.17582/journal.pjz/20191204121245

[ref56] Wilson E. O. (1984). Biophilia. Harvard University Press.

[ref57] World Health, A. (2019). "Eleventh revision of the international classification of diseases". (Geneva: World Health Organization).

[ref58] Wu J. Xiao M. Zhao Q. Wei S. Tian J. Luo Q. . (2018). Effects of horticultural therapy on quality of life and social function in patients with depression. Chin. Nurs. Manage. 18, 48–51. doi: 10.3969/j.issn.1672-1756.2018.01.013

[ref59] Xu Y. Ji Y. Su W. Zhang X. Yao F. (2022). Observation on effect of horticultural therapy on improvement of anxiety and depression behavior in patients with mild to moderate Alzheimer's disease. Chin. J. Alzheimer's Dis. Relat. Disord. 5, 300–303. doi: 10.3969/j.issn.2096-5516.2022.04.007

[ref60] Xu M. J. Lu S. Liu J. J. Xu F. (2023). Effectiveness of horticultural therapy in aged people with depression: a systematic review and meta-analysis. Front. Public Health 11:1142456. doi: 10.3389/fpubh.2023.1142456, PMID: 36969640 PMC10031070

[ref61] Yan W. Huang W. Wang Z. Cui S. (2017). To explore the effect of horticultural therapy on physical dysfunction and mental rehabilitation of stroke patients. Chin. J. Phys. Med. Rehabil. 39, 369–371. doi: 10.3760/cma.j.issn.0254-1424.2017.05.013

[ref62] Yang J. S. Zhang L. Y. Yang C. H. Li X. Y. Li Z. Q. (2024). Global, regional, and National Epidemiology of depression in working-age individuals, 1990-2019. Depress. Anxiety 2024:4747449. doi: 10.1155/2024/4747449, PMID: 40226639 PMC11919199

